# Bcl-2 protein frequency in patients with high-risk diffuse large B-cell lymphoma

**DOI:** 10.1590/S1516-31802010000100004

**Published:** 2010-01-07

**Authors:** Abrahão Elias Hallack, Sheila Aparecida Coelho Siqueira, Frederico Luiz Dulley, Alfredo Chauobah, Marcelo Belesso, Rosaura Saboia, Milton Artur Ruiz, Dalton Alencar Fischer Chamone, Juliana Pereira

**Affiliations:** I MD, PhD. Substitute professor, Department of Hematology, Universidade Federal de Juiz de Fora (UFJF), Juiz de Fora, Minas Gerais, Brazil.; II MD, PhD. Attending physician, Department of Pathological Anatomy, Faculdade de Medicina da Universidade de São Paulo (FMUSP), São Paulo, Brazil.; III MD, PhD. Associate professor, Department of Hematology, Faculdade de Medicina da Universidade de São Paulo (FMUSP), São Paulo, Brazil.; IV MD, PhD. Associate professsor, Department of Statistics, Universidade Federal de Juiz de Fora (UFJF), Juiz de Fora, Minas Gerais, Brazil.; V MD. Attending physician, Department of Hematology, Faculdade de Medicina da Universidade de São Paulo (FMUSP), São Paulo, Brazil.; VI MD, PhD. Titular professor, Department of Hematology, Faculdade de Medicina da Universidade de São Paulo (FMUSP), São Paulo, Brazil.

**Keywords:** Lymphoma, Oncogene proteins, Prognosis, Biological markers, Germinal center, Linfoma, Proteínas oncogênicas, Prognóstico, Marcadores biológicos, Centro germinativo

## Abstract

**CONTEXT AND OBJECTIVE:**

Gene expression and immunohistochemical profiling of diffuse large B-cell lymphoma (DLBCL) have revealed important prognostic subgroups: germinal center B-cell-like (GCB-like) DLBCL and activated B cell-like (ABC-like) DLBCL. Although few reports on high-risk DLBCL are available, the prognosis for the GCB-like subgroup has been shown to be better than that of the ABC-like subgroup. The role of Bcl-2 as a predictor of survival in DLBCL cases is unclear and its expression varies between the two subgroups of DLBCL. In this study, we analyzed the frequency and prognostic impact of Bcl-2 protein expression in high-risk DLBCL cases.

**DESIGN AND SETTING:**

Retrospective cohort study among DLBCL patients treated at Hospital das Clínicas, Faculdade de Medicina da Universidade de São Paulo (HC-FMUSP).

**METHODS:**

The prognostic impact of the expression of the proteins CD10, Bcl-6, MUM1 (multiple myeloma oncogene-1) and Bcl-2 on high-risk DLBCL cases was evaluated by means of immunohistochemistry. Seventy-three patients aged 18-60 years were evaluated for all these markers.

**RESULTS:**

Twenty-four cases (32.9%) were GCB-like and 49 (67.1%) were ABC-like, with no difference regarding complete remission, disease-free survival or overall survival rates. Twenty-seven patients (37%) showed Bcl-2 expression, which was the only independent factor predicting a worse prognosis for overall survival according to multivariate analysis.

**CONCLUSION:**

Bcl-2 protein was expressed in 37% of the high-risk DLBCL patients, without any difference between the ABC-like DLBCL and GCB-like DLBCL cases.

## RESUMO

**CONTEXTO E OBJETIVO:**

A expressão gênica e imunoistoquímica do linfoma difuso de grandes células B (LDGCB) vem permitindo a identificação de importantes subgrupos prognósticos: LDGCB do centro germinativo (CG) e LDGCB de células B ativadas (CBA). Entretanto, existem poucos dados disponíveis com LDGCB de alto risco, sendo o prognóstico dos LDGCB do CG melhor que os LDGCB de CBA. A participação do Bcl-2 como preditor de sobrevida nos LDGCB não é clara e sua expressão é variável entre os dois subgrupos de LDGCB. Neste estudo é avaliada a frequência e o prognóstico da expressão da proteína Bcl-2 em LDGCB de alto risco.

**TIPO DE ESTUDO E LOCAL:**

Estudo de coorte retrospectivo realizado entre portadores de LDGCB tratados no Hospital das Clínicas da Faculdade de Medicina da Universidade de São Paulo.

**MఙTODOS:**

Foi avaliado o impacto prognóstico da expressão das proteínas CD10, Bcl-6, MUM1 (multiple myeloma oncogene-1) e Bcl-2 por imunoistoquímica em LDGCB de alto risco. Foram avaliados, para todos os marcadores, 73 pacientes com idade de 18 a 60 anos.

**RESULTADOS:**

Vinte e quatro (32,9%) pacientes foram classificados como LDGCB do CG e 49 (67,1%) como LDGCB de CBA, sem diferença nas taxas de remissão completa, sobrevida livre de doença e sobrevida global. Vinte e sete (37%) apresentaram expressão de Bcl-2, o qual foi o único fator preditivo independente de pior prognóstico de sobrevida global à análise multivariada.

**CONCLUSÃO:**

A expressão da proteína Bcl-2 ocorreu em 37% dos portadores de LDGCB de alto risco, sem diferença entre os subgrupos de LDGCB do CG ou de CBA.

## INTRODUCTION

Diffuse large B-cell lymphoma (DLBCL) is a clinically and biologically heterogeneous disease that accounts for 30-35% of non-Hodgkin lymphoma (NHL) cases. About 58% of these cases are of high-intermediate or high international prognostic index (IPI) risk and 30-50% are cured with conventional cyclophosphamide, doxorubicin, vincristine and prednisone-like (CHOP-like) regimens.^1,2^ The gene expression shown by deoxyribonucleic acid (DNA) microarrays and by immunohistochemical staining has revealed different DLBCL prognostic subgroups according to the gene expression of malignant cells.^3-5^ Better prognosis has been demonstrated for germinal center B-cell-like (GCB-like) DLBCL cases than for activated B cell-like (ABC-like) DLBCL cases, regardless of IPI.^6^ However, most of these studies have included patients from different risk groups, whereas it is possible that most ABC-like DLBCL cases are from high-risk groups.^7^ Moreover, the prognosis for DLBCL cases presenting Bcl-2 expression remains unclear, and there are few studies on this protein in high IPI risk groups alone.^8,9^

## OBJECTIVE

To analyze the frequency and evolution of Bcl-2 expression in high-risk DLBCL patients according to the two subgroups.

## METHODS

### Patients and treatment

After obtaining approval from the Ethics Committee, a retrospective study was conducted. Between January 1992 and December 2005, 73 patients aged 18-60 years with high-intermediate or high risk DLBCL according to the aaIPI (age adjusted international prognostic index)^1,2^ were treated with CHOP or a CHOP-like protocol. Fourteen cases (19.1%) were consolidated with autologous bone marrow transplantation (ABMT) during the first complete remission. Patients who were human immunodeficiency virus (HIV) seropositive or who presented congestive heart failure, kidney failure or liver failure were not included in this study. Radiotherapy at a dose of 30 Gy was given to patients with bulky disease (i.e. > 10 cm) 28 days after the last chemotherapy or ABMT. Intrathecal chemotherapy (methotrexate 12 mg and dexamethasone 2 mg) was administered to patients with testicular disease or involvement of the facial bones. Refractory or relapsed disease was treated using the IVAC (ifosfamide, etoposide, high-dose cytarabine) protocol as salvage therapy.^10^

### Histology and immunohistochemical staining

All biopsies were reviewed by a hematopathologist at the Department of Pathological Anatomy of Hospital das Clínicas, Faculdade de Medicina da Universidade de São Paulo (HC-FMUSP). The GCB-like antigens Bcl-6 and CD10 and the ABC-like antigen MUM1 (multiple myeloma oncogene-1) were investigated by means of immunohistochemical reactions in order to define whether GCB-like DLBCL or ABC-like DLBCL was present.^11^ All the patients were also investigated regarding Bcl-2 antigens, by means of immunohistochemistry. We used mouse monoclonal antibodies against Bcl-6 (PIF6, dilution 1:40; Novocastra, Newcastle, United Kingdom), MUM1 (MUMIP, dilution 1:500; Dako, Glostrup, Denmark), CD10 (56C6, dilution 1:250; Novocastra, Newcastle, United Kingdom) and Bcl-2 (124, dilution 1:200; Dako, Glostrup, Denmark) as recommended by Hsu et al.^12^ Immunoreactivity of neoplastic cells greater than 10% was defined as positive for all markers.

### Response evaluation

All the patients underwent clinical and laboratory tests such as bilateral bone marrow biopsy and computed tomography of the neck, chest, abdomen and pelvis at diagnosis. The scans were repeated after the fourth chemotherapy cycle, at the end of the treatment and every three months for two years, and then every six months for a further three years. In cases of bone marrow disease at diagnosis, the bone marrow biopsy was repeated at the end of the treatment. We used Cheson's criteria for response evaluations.^13^

### Statistical analysis

Overall survival was calculated as the time interval between the date when the treatment began and either death or the last follow-up. Disease-free survival was defined as the time interval between complete remission and either first progression, relapse or death. For univariate analyses, the chi-square and Fisher exact tests were used to analyze the association between Bcl-2 and the categorical variables. The times until event endpoints were estimated in accordance with the Kaplan-Meier method. Cox's proportional hazards regression was used for multivariate analyses on overall survival and disease-free survival, using the variables that presented P values less than 0.05 from univariate analysis. The Statistical Package for the Social Sciences (SPSS) 13.0 software was used for statistical calculations, and P values less than 0.05 were considered statistically significant.

## RESULTS

### Clinical and immunohistochemical features

Table 1 shows the clinical characteristics of the 73 patients assessed by means of immunohistochemistry. Twenty-four (32.9%) of them presented GCB-like DLBCL and 49 (61.7%) ABC-like DLBCL, with similar distributions of clinical features in the two subgroups. Table 2 shows the patterns of CD10, Bcl-6, MUM1 and Bcl-2 antigen expression. There was no correlation between Bcl-2 expression and subgroups of GCB-like DLBCL and ABC-like DLBCL.

**Table 1.** Clinical data on the 73 patients evaluated

	GCB-like DLBCL		ABC-like DLBCL		Total		P*
	n	%	n	%	n	%
Age (years) Mean ± SD	42.21 ± 12.52	-	37.33 ± 12.66	-			0.718
Gender F	10	41.7	21	42.9	31	42.5	0.563
M	14	58.3	28	57.1	42	57.7	-
B symptoms	21	87.5	42	85.7	63	86.3	0.573
Extranodal	7	29.2	25	51	32	43.8	0.064
BM positive	4	16.7	12	24.5	16	21.9	0.33
Bulky	12	50	24	49	36	49.3	0.556
High LDH	22	91.7	44	89.8	66	90.4	0.582
Stage I/II	5	20.8	10	20.4	15	20.5	0.597
III/IV	19	79.2	39	79.6	58	79.5	-
aaIPI H-I	17	70.8	30	61.2	47	64.4	0.295
High	7	29.2	19	38.8	26	35.6	-
**Total**	**24**	**100**	**49**	**100**	**73**	**100**	-

GCB-like = germinal center B cell-like; DLBCL = diffuse large B-cell lymphoma; ABC-like = activated B cell-like; SD = standard deviation; F = female; M = male; B symptoms: recurrent fever (temperature, >38.3 °C [101 °F]), night sweats, or the loss of more than 10 percent of body weight; BM = bone marrow; LDH = lactate dehydrogenase; aaIPI = age-adjusted international prognostic index; H-I = high-intermediate.

* P < 0.05.

**Table 2.** Immunohistochemical staining results

	GCB-like DLBCL		ABC-like DLBCL		Total		P*
	n	%	n	%	n	%	
CD10	14	58.3	0	0	14	19.2	-
Bcl-6	20	83.3	16	32.6	36	49.3	-
MUM1	6	25	33	67.3	39	53.4	-
Bcl-2	9	37.5	18	36.7	27	37	0.574
**Total**	**24**	**32.8**	**49**	**67.1**	**73**	**89**	**-**

GCB-like = germinal center B cell-like; DLBCL = diffuse large B-cell lymphoma; ABC-like = activated B cell-like; MUM1 = multiple myeloma oncogene-1.

* P < 0.05 (chi-square test).

### Survival

For the full cohort of patients, the overall survival at 60 months was 42.8% (confidence interval, CI: 56-93) and the disease-free survival was 56.7% (CI: 63-110). However, the overall survival went down to 33.7% (CI: 35-83) for patients with bulky disease and went up to 53.4% (CI: 63-114) for patients without bulky disease (P = 0.03). Patients who achieved complete remission showed higher overall survival rates than did those who did not achieve complete remission with first-line therapy (P < 0.0001). Similarly, the disease-free survival (P = 0.015) among the ABMT group in first complete remission was higher than among patients who were kept under monitoring after complete remission.

The complete remission rate among the GCB-like DLBCL cases was 70.8% (CI: 67-74) and among the ABC-like cases, it was 61.2% (CI: 59-63) (P = 0.29). The overall survival among GCB-like cases at 60 months was 46.8% and among ABC-like cases, it was 43.3% (P = 0.885) (Table 3). The disease-free survival at 60 months was 60.8% for the GCB-like subgroup and 55.3% for the ABC-like subgroup (p = 0.365). Bcl-2 expression was shown in 27 patients (37%), and only 14 (51.8%) of them (CI: 48-55) achieved complete remission, while 33 (72%) (CI: 69-73) of Bcl-2 negative cases achieved complete remission (p = 0.073). Bcl-2 positive cases showed overall survival at 60 months of 16.4% (CI: 17-51.4), while Bcl-2 negative cases showed overall survival of 58.1% (CI: 71-119) (P = 0.004) (Table 3). The disease-free survival for Bcl-2 positive cases was 34% and for Bcl-2 negative cases, it was 65.6% (P = 0.12) (Table 3).

**Table 3.** Outcome according to immunohistochemical staining

	Complete remission		Overall survival		Disease-free survival	
	%	P	%	P	%	P
CD10 +	57.4	0.369	49	0.646	54.8	0.342
CD10 -	66.1	-	38.8	-	70	-
Bcl-6 +	49.3	0.128	43.1	0.85	60.5	0.816
Bcl-6 -	56.7	-	45.5	-	54.3	-
MUM1 +	64.1	0.576	46.8	0.95	58.4	0.795
MUM1 -	64.7	-	35.9	-	56.7	-
Bcl-2 +	51.8	0.073	16.4	0.004	34	0.12
Bcl-2 -	72	-	58.1	-	65.6	-
GCB	70.8	0.29	46.8	0.885	60.8	0.365
ABC	61.2	-	43.3	-	55.3	-

MUM1 = multiple myeloma oncogene-1; GCB = germinal center B cell-like; ABC = activated B cell-like.

### Multivariate analysis

In multivariate analysis, Bcl-2 positive cases showed a poor prognosis for overall survival (P = 0.01) with a relative risk (RR) of 2.44 (CI: 1.2-4.8). Likewise, the primary therapeutic response was poor (P < 0.0001), with RR of 7.36 (CI: 3.5-15.1). After excluding the primary response variable from the analysis, Bcl-2 expression remained an independent prognostic factor for survival (P = 0.006) with RR of 2.49 (CI: 1.3-4.7).

## DISCUSSION

Previous studies have identified two molecularly distinct subtypes of DLBCL that are indicative of different functional stages of B-cell differentiation.^4,14^ One type expresses the gene characteristics of GCB-like cells (GCB-like DLBCL), whereas the other type expresses genes that are normally seen during in vitro activation of peripheral blood B-cells (ABC-like DLBCL). Different prognoses for these two subgroups have been demonstrated, regardless of the IPI risk.^6,8,11,15^ More recently, Hans et al. have demonstrated similar results using immunohistochemical profiling, for other markers such as CD10, Bcl-6 and IRF4 (MUM1).^11^ Contrary to these studies, which evaluated patients with mixed IPI risk, we evaluated cases restricted to high-risk DLBCL that did not show survival differences between the GCB-like and ABC-like subgroups. Similarly, Nyman et al. demonstrated that this division does not have prognostic value when patients are treated with CHOP and the monoclonal antibody anti-CD20 (rituximab) (CHOP-R).^15^ These results show that prognostic factors change according to treatment, and according to the type of subgroups analyzed. They also show the heterogeneity of this disease.

On the other hand, there has not been any consensus regarding the prognostic value of CD10 antigen expression in DLBCL cases. Some authors have correlated it with better prognosis^9^ and others with worse prognosis. We did not show any correlation between CD10 expression and the likelihood of remission or survival, among high-risk DLBCL cases. Similarly, Bcl-6 antigen has been associated with good prognosis among patients treated with CHOP-like regimens, but not with CHOP-R^16^ or with sequential chemotherapy followed by ABMT in high-risk DLBCL cases.^17^

MUM1 (IRF4) has been demonstrated in post-GCB-like cases, and it is associated with ABC-like DLBCL and with worse prognosis. This subgroup of DLBCL has been associated with immunoblastic-like morphology and extranodal disease.^1^ In our report, 53.4% patients were positive for MUM1 expression, similar to the findings of Colomo.^18^ A study on high-risk patients who underwent sequential chemotherapy followed by ABMT showed that cutoff values lower than 70% positive expression of MUM1 cells did not have an impact on overall survival. On the other hand, when cutoff values higher than 70% positive expression of MUM1 were considered, favorable overall survival was observed for MUM1-negative cases.^17^ In the present study, we used a cutoff value of 10%, which may explain the lack of prognostic value for this marker in our study. The result from van Imhoff et al. suggests that the cutoff points for these markers should be determined according to their clinical significance for clinical application.

Iqbal et al. have suggested that the significance of Bcl-2 should be assessed within the context of DLBCL subgroups because of the controversy surrounding its prognostic significance.^14^ Some authors have correlated Bcl-2 expression with high-risk DLBCL.^17^ In our report, we observed Bcl-2 expression in 37% of our patients with high-risk DLBCL and we did not demonstrate any prognostic significance regarding the remission rate, even though the P-value was close to statistical significance (P = 0.07). However, there was prognostic significance with regard to overall survival (P = 0.004), as also reported by some other authors with similar cases.^19^ In multivariate analyses, Bcl-2 was shown to be an independent prognostic factor for survival (P = 0.01 and RR = 2.44), and these patients might benefit from protocols with greater aggressiveness, using rituximab-based chemotherapy.^20,21^

Our study is limited by its retrospective design with a small number of patients. Therefore, prospective studies with a larger number of patients are needed in order to evaluate the role of this protein in high-intermediate and high risk DLBCL cases among our population.

## CONCLUSION

Bcl-2 protein was expressed in 37% of the high-risk DLBCL cases, with no difference between the ABC-like DLBCL and GCB-like DLBCL subgroups. Bcl-2 expression was associated with worse overall survival among high-risk DLBCL cases.
